# Stop e-cigarette philanthropy: Amending the charity law to reinforce tobacco control in China

**DOI:** 10.18332/tid/177278

**Published:** 2024-01-24

**Authors:** Jiayi Jiang

**Affiliations:** 1 Law School, Central South University, Changsha, China

**Keywords:** China, tobacco control, e-cigarette, philanthropy, law reform

**Dear Editor,**

Article 13 of the World Health Organization’s Framework Convention on Tobacco Control (WHO FCTC) calls for a comprehensive ban on all forms of tobacco advertising, promotion, and sponsorship. Nevertheless, the tobacco industry often conducts philanthropic activities under the guise of corporate social responsibility (CSR) initiatives, including sponsoring education, scientific research, and cultural and social events to promote corporate image^1,2^.

In China, e-cigarettes are classified as tobacco products under the state’s tobacco monopoly strategy^3^. Our assessment of news reports and CSR reports from e-cigarette companies reveals that Chinese e-cigarette companies have diversified their philanthropic activities since the advertisement ban on e-cigarettes in 2019. Notably, many e-cigarette companies, independently or in collaboration with governments and non-governmental organizations, have made significant contributions. These include donations for preventing and controlling the COVID-19 pandemic^4^, earthquake relief^5^ and disaster response^6^, protection of biodiversity^7^, and infrastructure development for elementary schools or impoverished villages^8^.

However, the philanthropic activities of Chinese e-cigarette companies often serve a dual purpose. While they contribute to societal issues directly or indirectly, they also promote e-cigarette products or brands through news reports or their CSR reports on different platforms. By targeting vulnerable groups such as the impoverished and students, these activities can mislead recipients or other members of society into associating e-cigarette products or brands with charitable and positive attributes, potentially influencing public perceptions and consumer behavior.

In the context of China's Charity Law amendment in October 2023, many tobacco control advocates have emphasized the importance of fully implementing Article 13 of the WHO FCTC^9,10^. Currently, Article 40 of the Chinese Charity Law prohibits organizations and individuals from using charitable donations to advertise tobacco products. In response to the challenges posed by tobacco philanthropy, our first recommendation is to prohibit the philanthropic activities of e-cigarette companies. This is critical for bringing e-cigarettes under the same regulatory controls as traditional tobacco products. Our second recommendation is to prohibit direct and indirect donations that could result in covert advertising, thus preventing tobacco producers and sellers from using philanthropy for marketing purposes. These amendments are crucial for strengthening tobacco control measures in China and globally, particularly in closing existing gaps that e-cigarette companies exploit, thereby aligning public health policies with the WHO FCTC.

## Data Availability

Data sharing is not applicable to this article as no new data were created.
